# Local IL-17 Production Exerts a Protective Role in Murine Experimental Glomerulonephritis

**DOI:** 10.1371/journal.pone.0136238

**Published:** 2015-08-28

**Authors:** Sally Hamour, Poh-Yi Gan, Ruth Pepper, Fernanda Florez Barros, Hsu-Han Wang, Kim O’Sullivan, Yoichiro Iwakura, Terence Cook, Charles Pusey, Stephen Holdsworth, Alan Salama

**Affiliations:** 1 UCL Centre for Nephrology, Royal Free Hospital, London, United Kingdom; 2 Centre for Inflammatory diseases, Monash University, Melbourne, Australia; 3 Research Institute for Biomedical Sciences, Tokyo University of Science, Tokyo, Japan; 4 Centre for Inflammation and Complement Research, Department of Medicine, Imperial College London, London, United Kingdom; 5 Renal Section, Department of Medicine, Imperial College London, London, United Kingdom; Radboud university medical center, NETHERLANDS

## Abstract

IL-17 is a pro-inflammatory cytokine implicated in the pathogenesis of glomerulonephritis and IL-17 deficient mice are protected from nephrotoxic nephritis. However, a regulatory role for IL-17 has recently emerged. We describe a novel protective function for IL-17 in the kidney. Bone marrow chimeras were created using wild-type and IL-17 deficient mice and nephrotoxic nephritis was induced. IL-17 deficient hosts transplanted with wild-type bone marrow had worse disease by all indices compared to wild-type to wild-type bone marrow transplants (serum urea p<0.05; glomerular thrombosis p<0.05; tubular damage p<0.01), suggesting that in wild-type mice, IL-17 production by renal cells resistant to radiation is protective. IL-17 deficient mice transplanted with wild-type bone marrow also had a comparatively altered renal phenotype, with significant differences in renal cytokines (IL-10 p<0.01; IL-1β p<0.001; IL-23 p<0.01), and macrophage phenotype (expression of mannose receptor p<0.05; inducible nitric oxide synthase p<0.001). Finally we show that renal mast cells are resistant to radiation and produce IL-17, suggesting they are potential local mediators of disease protection. This is a novel role for intrinsic cells in the kidney that are radio-resistant and produce IL-17 to mediate protection in nephrotoxic nephritis. This has clinical significance as IL-17 blockade is being trialled as a therapeutic strategy in some autoimmune diseases.

## Introduction

IL-17 is a pro-inflammatory cytokine with varied roles in inflammation, host defence and autoimmunity [1, 2]. There has been renewed interest in its functions since the discovery of the Thelper (Th)17 cell phenotype [3, 4], a distinct Th cell lineage characterised by the production of IL-17. Th17 cells are now recognised as central to the pathogenesis of autoimmunity. In immune-mediated kidney injury, mice completely deficient in IL-17 or the related cytokine IL-23 are protected from renal disease [5–8].

Recently, a more nuanced perspective of the role of IL-17 in autoimmunity has emerged and there is evidence for IL-17 mediating protection from disease in graft versus host disease [9] and asthma [10]. In experimental models of colitis the evidence for a protective role for IL-17 varies depending on the model used [11, 12]. In the CD45RBhi model, transfer of IL-17^-/-^or IL-17RA^-/-^ CD45RBhi cells into RAG^-/-^ mice resulted in worse disease than transfer of WT cells, demonstrating that IL-17 can be protective [13]. In experimental autoimmune uveitis, treatment with anti-IL-17 antibodies blocked disease development but injection of uveitis-susceptible rats and mice with recombinant human IL-17 unexpectedly ameliorated disease [14]. In autologous (non-accelerated) nephrotoxic nephritis (NTN), IL-17 promotes early kidney injury at day 6 but by day 21, IL-17 attenuates established disease [15]. Thus, although IL-17 was initially recognised a pro-inflammatory cytokine, it can contribute to both the induction and suppression of immune-mediated inflammation. Although these functions might appear to be contradictory, there are many other biological examples such as the role of IL-2 in T-cell proliferation and differentiation in contrast to its role in activation-induced cell death.

We hypothesised that transplantation of WT bone marrow to IL-17 deficient mice would restore disease susceptibility in NTN. This hypothesis was upheld but we also demonstrate a novel protective function for local IL-17 production in this model with important implications for translating IL-17 blockade to the clinical setting.

## Materials and Methods

### Mice

IL-17^-/-^ mice were provided by Professor Iwakura, University of Tokyo [16]. Homozygous mice were bred to provide experimental mice at the Central Biological Services Unit at Hammersmith Hospital. Control age- and sex- matched C57BL/6 mice were obtained from Charles River laboratories. Mice were 8–12 weeks of age, housed with free access to food and water and kept in a specific pathogen free environment according to institutional guidelines. All procedures were performed in accordance with the United Kingdom Animals (Scientific Procedures) Act, 1986. All the procedures were approved by the Home Office UK. ARRIVE Guidelines Checklist in S1 ARRIVE Checklist.

Kit^W-sh/W-sh^ were bred and housed in specific pathogen free conditions at Monash Medical Centre Animal Facilities, Monash University, Australia. Experiments conducted were approved by Monash University Animal Ethics Committee in accordance with the Australian National Health and Medical Research Council animal experimentation guidelines.

### Generation of bone marrow chimeras

Recipient C57BL/6 or IL-17^-/-^ female mice aged 6–8 weeks were lethally irradiated with a dose of 8Gy and immediately injected with 10 million donor bone marrow cells via the tail vein. Mice were housed in individually ventilated cages with free access to autoclaved food and acidified water. After 8 weeks, genetic reconstitution was determined by genotyping by PCR for WT and IL-17 deficient alleles on DNA extracted from blood.

### Induction of accelerated NTN

Mice were pre-immunised subcutaneously with 0.2mg of sheep IgG (Sigma-Aldrich) in a mix with Complete Freund’s Adjuvant (Sigma Aldrich). Five days after immunisation, the animals were injected with 200μl diluted sheep nephrotoxic globulin, in 0.9% NaCl via the tail vein. Mice were humanely killed 8–9 days after NTS injection, before harvesting kidneys, spleen, and blood. Blood was allowed to clot at room temperature before separating serum which was stored at -80°C.

### Assessment of renal function: Measurement of proteinuria, urea, creatinine

Individual 24-hour collections of urine were performed using metabolic cages with free access to food and water. The concentration of protein in the urine of the mice was assessed using a Sulphosalicylic acid assay. Urea and creatinine were measured in the Department of Clinical Chemistry, Hammersmith hospital.

### Histological assessment

All microscopic analysis was performed blinded to sample identity. Glomerular thrombosis was assessed by scoring individual glomeruli on PAS-stained kidney sections for PAS positive material. Tubular damage was assessed over the entire section for the presence of tubular dilatation and tubular casts. Sections were scored from 0–4 based on the total area of the section involved.

### Immunofluorescence

Direct IF on frozen sections was used to detect mouse and sheep IgG on sections of mouse kidney using goat anti-mouse IgG FITC (Sigma) or monoclonal anti-goat/sheep IgG FITC (Sigma). Fluorescence for each glomerulus was scored using a visual fluorescence scale from 0–3. 25 glomeruli were scored for each section. All sections from one experiment were stained at the same time and analysed in the same session.

### Immunohistochemistry

Immunoperoxidase staining to detect CD68 positive macrophages and CD4+ T-cells was performed on frozen PLP-fixed sections using the Polink-2 HRP Plus kit (Newmarket Scientific). Primary antibodies used were rat anti-mouse CD68 (Serotec) and rat anti-mouse CD4 (Pharmingen). For intra-glomerular scoring, only macrophages or CD4+ cells inside glomeruli, as defined by Bowman’s capsule, were counted. 25 glomeruli were scored per kidney. For interstitial macrophage and CD4+ cell scoring, whole sections were inspected and all kidneys from a single experiment were ranked in order of increasing interstitial macrophage or CD4+ staining. Sequential ascending scores were then assigned for all sections.

### ELISA for serum mouse anti-sheep IgG

Total mouse anti-sheep IgG and IgG sub-classes were measured in mouse serum by ELISA at the end of the experiment. For total IgG, ELISA plates were coated overnight with 100μg/ml of sheep IgG (Sigma). The secondary antibody used was Fc-specific goat anti-mouse IgG conjugated to alkaline phosphatase (Sigma). For determination of IgG subclasses, goat anti-mouse IgG1, IgG2a, IgG2b, IgG2c, IgG3 (all Southern Biotechnology, Birmingham, Alabama, USA) were used. Plates were developed using p-nitrophenyl phosphate (Sigma) before determination of the optical density by the ELISA plate reader at 405nm.

### Quantitative PCR

RNA from whole kidney was extracted using Trizol and re-extracted for purity. Quality was verified by spectrophotometry and gel electrophoresis. Complementary DNA was synthesised from RNA using SuperScript III Reverse Transcriptase, Oligo(dT)12-18, 10mM dNTP mix, 5x1st strand buffer, 0.1M Dithiothreitol and RNAse out (all Invitrogen), with reverse-transcriptase negative and blank (H2O) controls. Quantitative real-time PCR from cDNA was carried out using a Mesa Blue qPCRMastermix plus for SYBR assay (Eurogentec Ltd, Southampton, UK) on a Mastercycler realplex2 (Eppendorf). All samples were assayed in triplicate. cDNA from 3 normal WT kidneys was used as a control. For each primer pair, mRNA abundance was normalised to the amount of GAPDH, determined using the ΔΔCt method.

Primer sequences are as follows:

GAPDH F: 5’GGGTGTGAACCACGAGAAAT-3’ R: 5’GTCTTCTGGGTGGCAGTGAT-3’;

IL-17 F: 5'ACCGCAATGAAGACCCTGAT-3' R: 5'TCCCTCCGCATTGACACA -3';

IL-10 F: 5’GGTTGCCAAGCCTTATCGGA-3’ R: 5’ACCTGCTCCACTGCCTTGCT-3’;

IL-1β F: 5’AACCTGCTGGTGTGTGACGTTC-3’ R: 5’CAGCACGAGGCTTTTTTGTTGT-3’;

IFNγ F: 5’ATCTGGAGGAACTGGCAAAA-3’ R: 5’TTCAAGACTTCAAAGAGTCTGAGG-3’;

IL-23p19 F: 5′-TGTGCCCCGTATCCAGTGT-3’ R: 5′-CGGATCCTTTGCAAGCAGAA-3′;

IL-4 F: 5'ACAGGAGAAGGGACGCCAT-3' R: 5'GAAGCCCTACAGACGAGCTCA-3';

TGFβ F: 5’TGAGTGGCTGTCTTTTGACG-3’ R: 5’AGCCCTGTATTCCGTCTCCT-3’[17];

Mannose Receptor F: 5’CAAGGAAGGTTGGCATTTGT-3’ R: 5’CCTTTCAGTCCTTTGCAAGC-3’[18];

Arginase F: 5’CAGAAGAATGGAAGAGTCAG-3’ R: 5’CAGATATGCAGGGAGTCACC-3’[18];

iNOS F: 5’CCAAGCCCTCACCTACTTCC-3’ R:5’CTCTGAGGGCTGACACAAGG-3’[18];

Tbet F: 5’CCTGGACCCAACTGTCAACT-3’ R: 5’AACTGTGTTCCCGAGGTGTC-3’[19].

### FACS analysis of IL-17 receptor expression in wild-type and IL-17 deficient mice

Spleens from healthy WT and IL-17 deficient mice were collected in sterile Falcon tubes containing PBS (Sigma) and processed individually to obtain single cell suspensions. Briefly, spleens were mashed through two cell strainers (70μm and 40μm) and rinsed with PBS (Sigma). Suspended cells were transferred to a 50ml Falcon tube and spun at 350xg. RBC were lysed by re-suspending cell pellets in 1X RBC Lysis Buffer (Biolegend). Cell viability was assessed using the trypan blue exclusion test, and cells were counted using a haemocytometer.

Flow cytometric analysis of IL-17 receptor expression was performed using Anti-Mouse CD217 (IL-17 Receptor A) PE (Cat. no 12-7182-80, eBioscience) and Rat IgG2a Isotype Control PE (Cat. no 12-4321-73, eBioscience) on a BD LSRFortessa cytometer. Staining was performed for 20 minutes followed by washing. For the analyses, samples were gated on splenocyte population based on size, as assessed by SSC and FSC. Analysis was performed using FlowJo analysis software.

### Renal leukocytes: Resistance to radiation and production of IL-17

Mast cells were differentiated in vitro from bone marrow. Cells were harvested from 6 to 8 week old WT C57BL/6 mice. Cells were cultured in RPMI supplemented with 15% FCS, 1% Pen/Strep, 2mM L-glutamine, 1mM Sodium pyruvate (Invitrogen, Melbourne, Australia), 50μM 2-mercaptoethanol (Sigma-Aldrich), IL-3 (obtained from WEHI3 cell culture supernatants) and 12.5ng/ml recombinant stem cell factor (R&D Systems, Minneapolis, MN). Non-adherent cells were transferred into fresh culture media twice weekly for 6–8 weeks. Mast cell purity (>98%) was assessed using toluidine blue stain (1% toluidine blue [Sigma] in 50% ethanol, pH 2.3).

5x10^6^ bone marrow-derived mast cells were injected under the renal capsule of mast cell deficient Kit^W-sh/W-sh^mice while on the opposite side of the same kidney, 5x10^6^splenocytes (from Ly5.1 mice) were injected under the renal capsule. Twenty four hours after injection, Kit^W-sh/W-sh^micereceived 800-rad total body irradiation (n = 6 and n = 8 non-irradiated control Kit^W-sh/W-sh^mice). 24hourslater, 10ug of LPS (Sigma-Aldrich) was injected intra-peritoneally to stimulate mast cells to produce IL-17A. Mice were humanely killed 4 hours post LPS administration.

### Confocal Microscopy

Snap-frozen sections were cut at 6μm and fixed in acetone for 10 minutes. Sections were blocked for 30 minutes in 10% chicken sera diluted in 5% BSA/PBS, and directly conjugated with primary antibodies 2μg/ml rat anti-mouse C-kit-R-PE (BD Pharmingen San Jose, CA, USA), 8μg/ml rat anti-mouse IL17A-FITC (BD Pharmingen) and 4μg/ml rat anti mouse CD45.1- BD Horizon (V450)(BD Pharmingen) diluted in 1%BSA/PBS for 1 hour, washed in PBS and mounted with antifade mounting media (DAKO, Glostrup, Denmark). Images were taken on a Nikon Ti-E inverted microscope attached to a Laser scanning head. Single plane 512x512x12 bit images were captured in a line sequential manner using a 20x 0.75 objective with pinhole set a 1.0AU. C-kit, IL17A and CD45.1 IF from the renal capsule (10 fields) was measured using area x intensity x density in Image J analysis software (National Institute of Health, Bethesda, Maryland, USA), and averaged per sample and represented as arbitrary units (AU).

### Statistical analysis

All statistics were performed using GraphPad prism 4.0 (GraphPad Software, San Diego California, USA). Non-parametric tests of significance were applied throughout. For comparing 2 groups, Mann-Whitney U test was used. One way analysis of variance (ANOVA) used non-parametric Kruskal-Wallis test and Dunn’s multiple comparison post-test for groups of three or more. Two-way ANOVA was used to analyse differences between groups from more than one experiment. Two tailed analysis was carried out with significance defined as P<0.05 with 95% confidence.

## Results

### IL-17^-/-^ mice are protected from nephrotoxic nephritis

**Fig 1:**
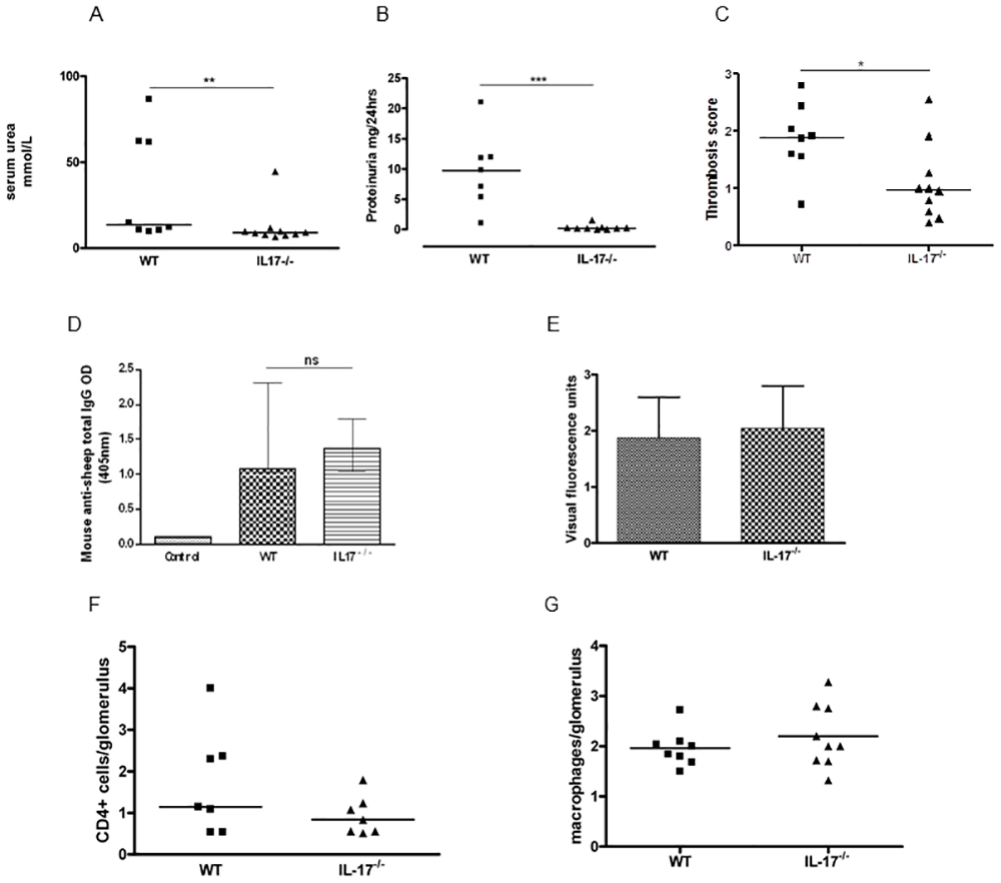
IL-17^-/-^ mice are protected from NTN. IL17^-/-^ mice had significantly (A) lower serum urea (p<0.01), (B) less proteinuria (p<0.001) and (C) less thrombosis (p<0.05) compared to WT mice. There was no difference in humoral responses between the groups either for (D) total serum mouse anti-sheep IgG or (E) glomerular deposition of mouse anti-sheep IgG. There was no significant difference in cellular infiltration of glomeruli by either (F) CD4+ T cells or (G) CD68+ macrophages. Analysis by Two-Way ANOVA and non-parametric Mann-Whitney U test. Bars represent median and range. Lines represent median. * = p<0.05; ** = p<0.01; *** = p<0.001. Differences are non-significant unless otherwise stated. Data shows combined results from 2 experiments of at least 4 mice per group.

In this model, mice develop a proliferative and thrombotic glomerulonephritis by 7 days. The early phase is characterised by neutrophil infiltration whilst later on, CD4+ T-cells and macrophages predominate [20, 21]. Mice deficient in IL-17 are protected from accelerated NTN 8–9 days following disease induction in keeping with work by other groups [6]. No difference in humoral responses (mouse anti-sheep circulating or deposited IgG) or intra-glomerular cellular infiltration of macrophages or CD4+ T cells was found at this time point (Fig 1).

### In IL-17^-/-^ mice, disease susceptibility is restored by transplantation of WT bone marrow and results in significantly more severe disease compared to WT to WT bone marrow transplant mice

**Fig 2:**
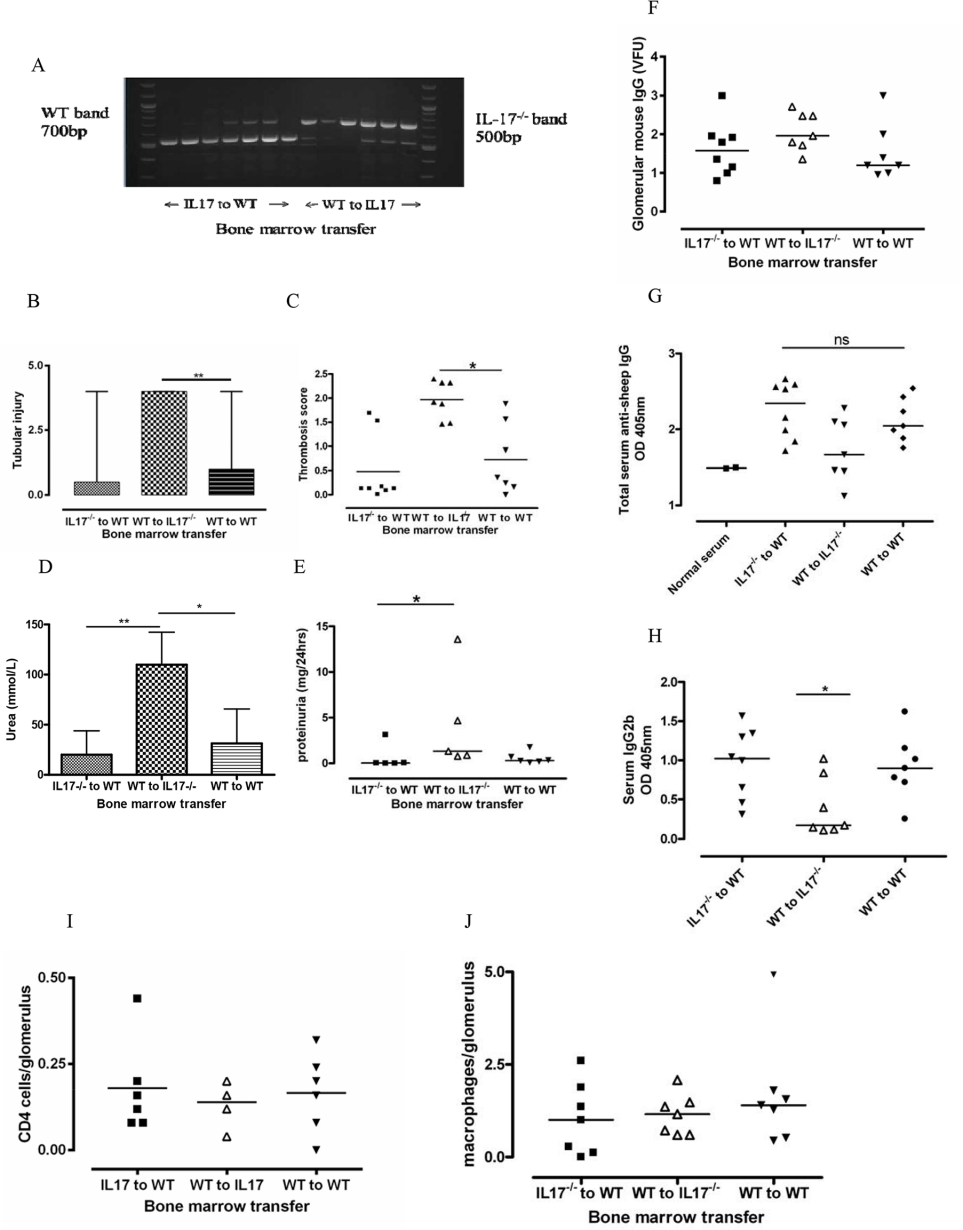
Transplantation of WT bone marrow to IL-17^-/-^ mice restores and augments disease susceptibility. (A) Haematopoietic reconstitution of bone marrow transplant mice. DNA extracted from blood was analysed by PCR for WT and IL-17^-/-^ alleles. All mice reconstituted >90% donor bone marrow. WT to IL-17^-/-^ mice have (B) significantly greater tubular injury (p<0.01) and (C) more glomerular thrombosis (p<0.05) than WT to WT mice. Renal function is worse as measured by serum urea (D) than the other two groups. WT to IL-17^-/-^ mice had more proteinuria than IL-17^-/-^ to WT mice (p<0.05) (E), but not statistically more than WT to WT mice due to small numbers, many of the severely affected mice were oligo-anuric and produced insufficient urine to quantify. Humoral immune responses and infiltrating cell numbers were similar between the groups despite differences in disease outcomes. (F) There was no difference in glomerular mouse IgG between the groups. (G) Serum showed no differences in total anti-sheep IgG produced. (H) There was significantly less IgG2b in WT to IL-17^-/-^ mice in the first experiment and a similar tendency in the second experiment. There was no difference in numbers of (I) CD4+ T cells or (J) CD68+ macrophages in the glomeruli. Analysis by Two-Way ANOVA and non-parametric Mann-Whitney U test. Bars represent median and range. Lines represent median. * = p<0.05; ** = p<0.01; *** = p<0.001. Representative data shown from 1 experiment of 2 separate experiments of at least 6 mice per group.

In order to characterise the contribution of intrinsic and bone marrow-derived IL-17 to the pathogenesis of NTN we performed transplantation of WT (C57BL/6) bone marrow into WT and IL-17 deficient recipients, and transplanted IL-17 deficient bone marrow into WT recipients. After 8 weeks, all mice had >90% haematopoietic reconstitution (Fig 2a). Accelerated NTN was induced using our standard protocol.

**Fig 3:**
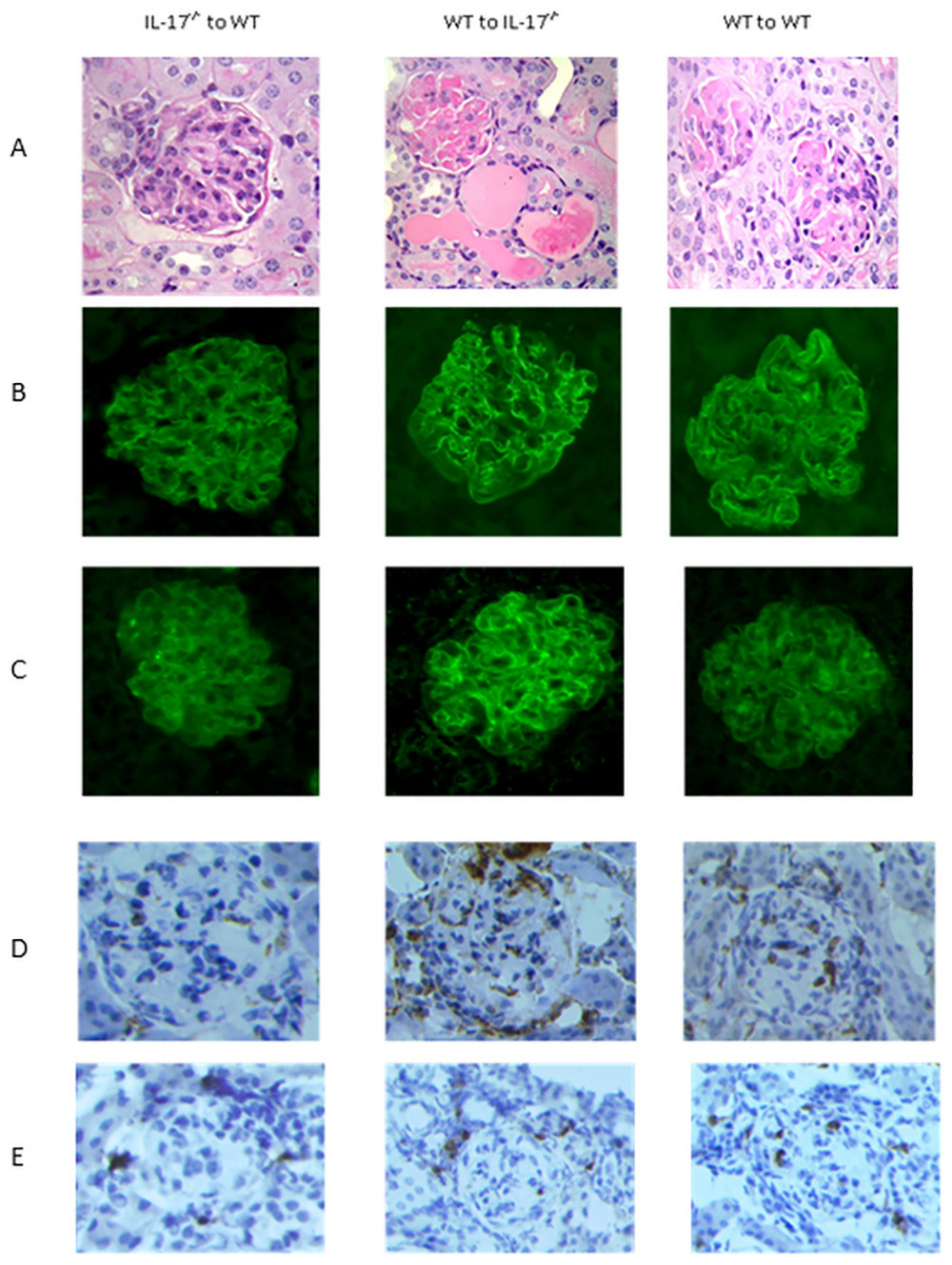
Representative histological sections from the 3 bone marrow transplant groups with NTN. (A) PAS-stained sections of renal histology. The WT to IL-17^-/-^ group have severe glomerular thrombosis with profound tubular dilatation and casts, significantly more than the other groups. (B) and (C) Immunofluorescence for glomerular deposition of sheep and mouse IgG respectively. There was no difference between the groups. (D) and (E) Representative glomerular staining for CD68 and CD4 respectively. Magnification x400.

Unexpectedly, disease susceptibility in IL-17 deficient mice transplanted with WT bone marrow was significantly more severe than their WT to WT counterparts. PAS-stained kidney sections at day 8 showed profound focal glomerular and tubular damage. There was glomerular thrombosis with massive tubular dilatation and casts (Fig 3a). This group had significantly more renal injury as measured by tubular injury (median(range): IL-17^-/-^ to WT 0.5 (0.0–4.0); WT to IL-17^-/-^ 4.0 (2.0–4.0); WT to WT 1.0 (0.00–4.0) p<0.01, analysis by one way ANOVA) and glomerular thrombosis score (median(range): IL-17^-/-^ to WT 0.12 (0.00–1.68); WT to IL-17^-/-^ 1.92 (1.46–2.40); WT to WT 0.36 (0.00–1.88); p<0.05) (Fig 2b and 2c). Frequency of crescents was low in all groups (data not shown). WT mice transplanted with IL-17^-/-^ bone marrow had the mildest disease although they were not completely protected.

Renal function as measured by serum urea was also significantly worse in the WT to IL-17 deficient transplant group compared to WT to WT mice (p<0.05) and IL-17 deficient to WT mice (p<0.01). Values for urea (median (range)) were as follows: (IL-17^-/-^ to WT 12.2 (6.4–65.8); WT to IL-17^-/-^ 109.8 (94.4–151.0); WT to WT 12.4 (8.1–80.0)) (Fig 2d). Levels of creatinine did not vary significantly between groups (data not shown). Levels of proteinuria in the WT to IL-17 deficient transplant group were higher than in the IL-17 deficient to WT transplant group (median(range): IL-17^-/-^ to WT 0.04 (0.00–3.13); WT to IL-17^-/-^ 1.33 (0.78–13.59); WT to WT 0.30 (0.09–1.77); p<0.05) (Fig 2e). They also tended to be higher than the WT to WT transplant group, but did not reach statistical significance as some of the WT to IL-17^-/-^ mice with severe disease were oliguric and produced insufficient urine to quantify.

### Humoral immune responses do not differ significantly between experimental groups in the bone marrow transplant mice despite differences in disease outcome

IL-17 has a role in humoral immunity and IL-17 deficient mice have been characterised as having lower levels of total IgG and IgG subclasses [16]. In order to assess the humoral immune response at the end point of the experiment, we examined glomerular deposition of sheep and mouse IgG and serum levels of mouse anti-sheep IgG. There was no difference in glomerular deposition of total sheep IgG (Fig 2f) or mouse IgG (data not shown) between the groups by immunofluorescence (IF). By serum ELISA for mouse anti-sheep IgG, WT-IL-17^-/-^ mice had a tendency to lower total serum total IgG (Fig 2g) and IgG1. WT-IL-17^-/-^ mice had less IgG2b (median(range): IL-17^-/-^ to WT 1.02 (0.32–1.57); WT to IL-17^-/-^ 0.18 (0.11–1.02); WT to WT 0.90 (0.26–1.62); p<0.05) in the first experiment (Fig 2h)and followed a similar trend in the second experiment. There were no significant differences for any of the other subclasses (p>0.05).

### Numbers of infiltrating macrophages and CD4+ cells do not differ between the experimental groups despite differences in disease outcome

In order to assess the cell-mediated immune response, we quantified the number of intra-glomerular macrophages and CD4+ cells by immunoperoxidase staining on PLP-fixed frozen sections at the end point of the experiment and scored the interstitium for cellular infiltration. There was no difference in numbers of intra-glomerular CD4+ cells or macrophages between the groups (CD4+ cells: median(range): IL-17^-/-^ to WT 0.14 (0.08–0.44); WT to IL-17^-/-^ 0.14 (0.04–0.20); WT to WT 0.18 (0.00–0.32); p>0.05); (Macrophages: median(range): IL-17^-/-^ to WT 1.0 (0.0–2.6); WT to IL-17^-/-^ 1.2 (0.6–2.1); WT to WT 1.4 (0.4–5.0); p>0.05) (Fig 2i and 2j). Numbers of interstitial macrophages and CD4+ cells tended to be higher in the WT-IL-17^-/-^ group but this did not reach statistical significance (data not shown).

### WT to IL-17^-/-^ transplant mice, the most severely affected group, had an altered renal cytokine profile with less IL-10, less IL-1β and less IL-23

Quantitative PCR was carried out on a number of important immune mediators: TGFβ, IL-1β, IL-17, IL-23 (Th17 axis and development); IFNγ and Tbet (Th1 axis); IL-4 (Th2 axis) and IL-10 (immuno-regulatory).

**Fig 4:**
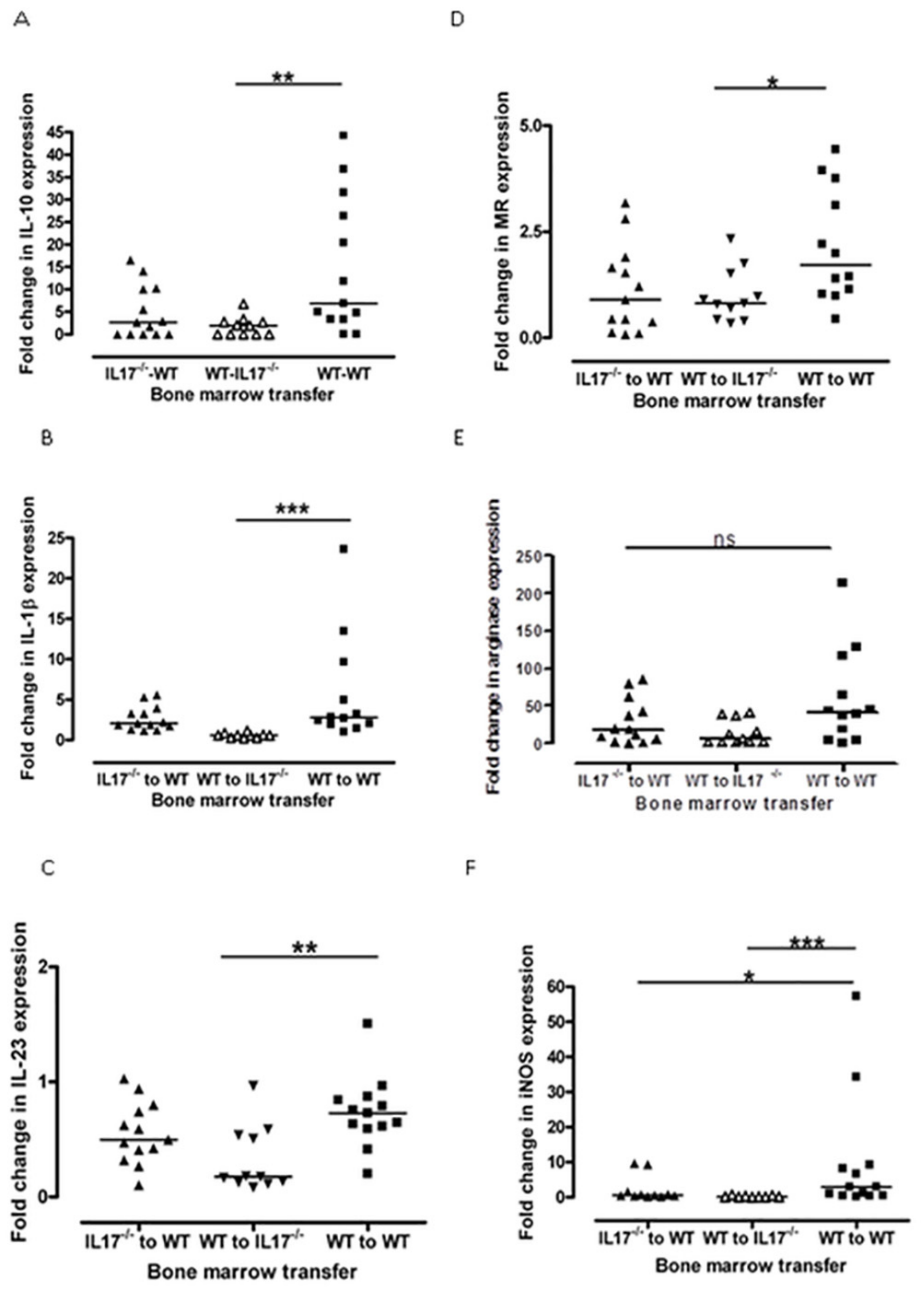
There were significant differences in cytokine levels and macrophage phenotype between the groups. The most severely affected group, WT to IL-17^-/-^ bone marrow transplants had significantly less renal (A)IL-10, (B)IL-1β, (C) IL-23 by qPCR compared to WT to WT transplant mice. They also had a different macrophage phenotype with significantly less (D) mannose receptor and (F) iNOS. Although arginase levels had a tendency to be lower in WT to IL-17^-/-^ bone marrow transplants, this was not statistically significant (E). Analysis by Two-Way ANOVA and non-parametric Mann-Whitney U test. Lines represent median. * = p<0.05; ** = p<0.01; *** = p<0.001. Data shows combined results from 2 separate experiments of at least 6 mice per group.

WT to IL-17 deficient transplant mice had significantly less IL-10 (median(range): IL-17^-/-^ to WT 2.7 (0.0–16.6); WT to IL-17^-/-^ 1.9 (0.0–6.8); WT to WT 6.9 (0.0–44.3); p<0.01); significantly less IL-1β (median(range): IL-17^-/-^ to WT 2.1 (1.2–5.6); WT to IL-17^-/-^ 0.6 (0.2–1.2); WT to WT 2.8 (1.0–23.6); p<0.001) and significantly less IL-23 (median(range): IL-17^-/-^ to WT 0.5 (0.1–1.0); WT to IL-17^-/-^ 0.2 (0.1–1.0); WT to WT 0.7 (0.2–1.5); p<0.01), compared to WT to WT transplant mice (Fig 4a, 4b and 4c). There was no difference in levels between the groups of any of the other cytokines or transcription factors.

### WT-IL-17^-/-^ transplant mice had a different macrophage phenotype with less mannose receptor and less iNOS

Mannose receptor and arginase are associated with an ‘alternatively activated’ or more reparative macrophage phenotype whilst expression of iNOS is associated with classically activated ‘pro-inflammatory’ macrophages, reviewed in [22, 23]. The WT to IL-17 deficient group had less mannose receptor than WT to WT transplant mice (median(range): IL-17^-/-^ to WT 0.9 (0.1–3.2); WT to IL-17^-/-^ 0.8 (0.3–2.3); WT to WT 1.7 (0.4–4.4); p<0.05). Although arginase levels had a tendency to be lower in the WT into IL-17 deficient bone marrow transplant group, this did not reach statistical significance. There was less iNOS in the WT to IL-17 deficient group compared to the WT to WT group (median(range): IL-17^-/-^ to WT 0.6 (0.2–9.7); WT to IL-17^-/-^ 0.1 (0.0–0.7); WT to WT 3.0 (0.2–57.3); p<0.001) (Fig 4d, 4e and 4f).

### Levels of IL-17 did not vary significantly between the experimental groups

**Fig 5:**
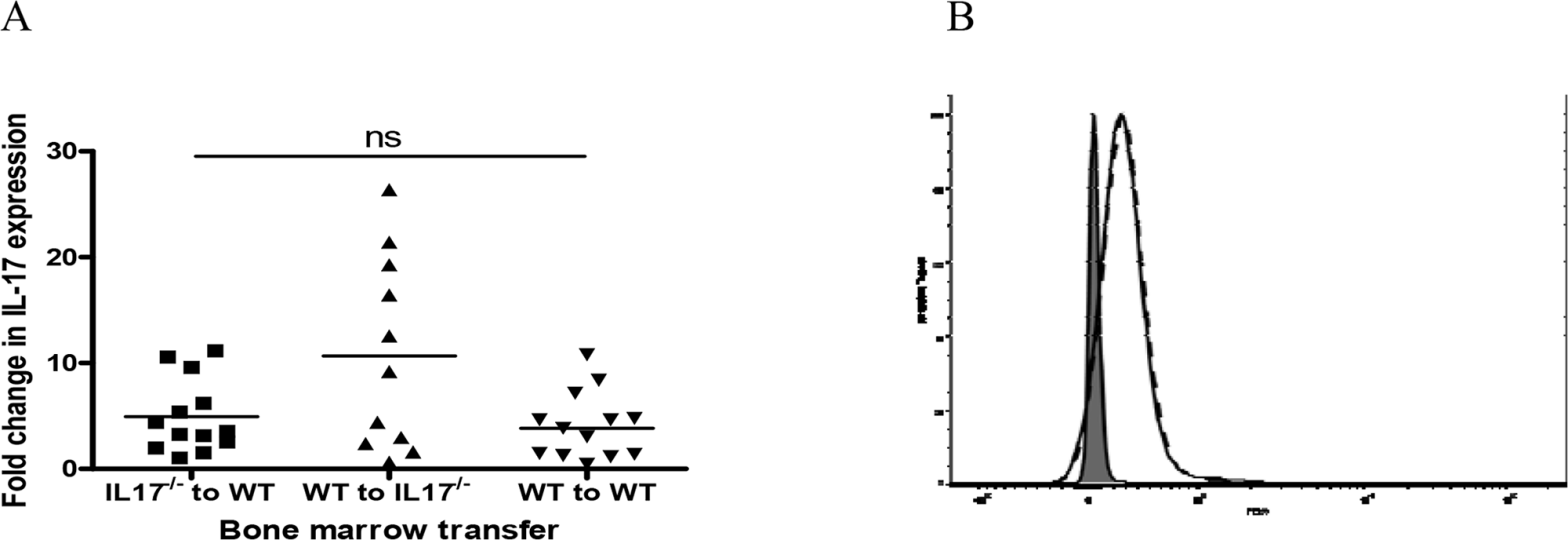
(A) Levels of renal IL-17 by qCR were not different between the 3 experimental groups. (B) Flow cytometry analysis of IL-17 receptor expression in splenocytes from unmanipulated WT and IL-17^-/-^ mice. Shaded area represents PE isotype control. Dotted line represents WT splenocytes and superimposed solid line represents IL-17^-/-^splenocytes. There was no difference in expression of the IL-17 receptor in splenocytes from healthy WT and IL-17^-/-^ mice (B).

There was no significant difference in levels of IL-17 between the experimental groups at this time point, including the WT group transplanted with IL-17 deficient haematopoietic cells, which underscores the importance of IL-17 production by intrinsic cells in this model (Fig 5a). Furthermore there was no difference in expression of the IL-17 receptor in splenocytes from healthy WT and IL-17^-/-^mice (Fig 5b), and so over-expression of the IL-17 receptor cannot explain increased disease in the IL-17 deficient mice transplanted with WT bone marrow.

### Mast cells continue to produce IL-17 after irradiation potentially mediating disease protection in this model

**Fig 6:**
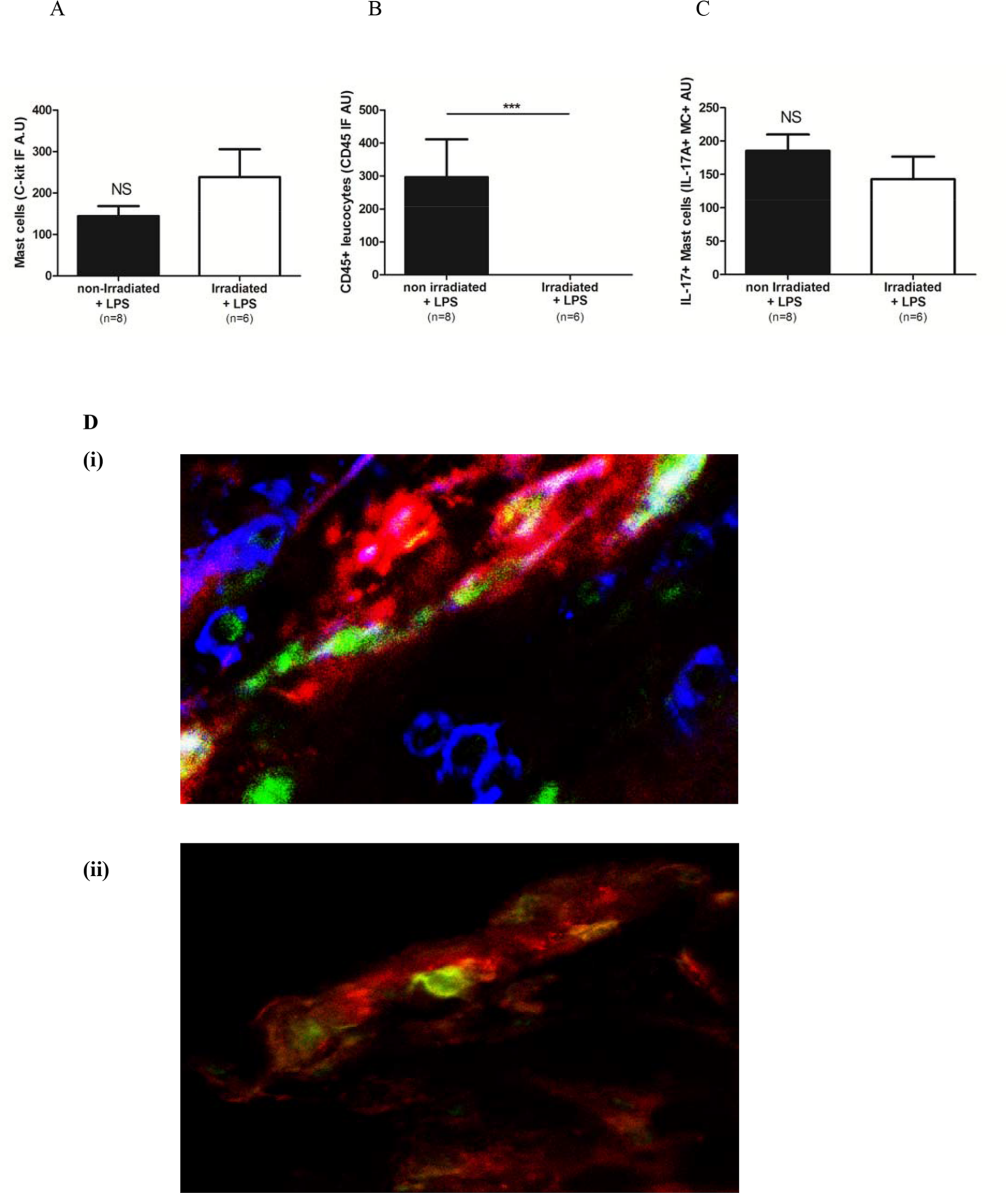
Mast cells persist following irradiation and produce IL-17. Mast cells persist following irradiation and retain the ability to produce IL-17 following stimulation with LPS. (A) Numbers of intra-renal mast cells are unchanged following irradiation. (B) In contrast, CD45+ splenocytes are completely removed by irradiation. (C) Production of IL-17 by mast cells is unaffected by irradiation. (D) Confocal microscopy images from renal cortex. (i) In the absence of irradiation, mast cells (red), CD45+ splenocytes (blue) and IL-17 (green) are demonstrated. (ii) Following irradiation, CD45+ leucocytes are absent. Mast cells and IL-17 production are unaffected and co-localise (yellow). X1200. Data presented are representative of two independent experiments of at least 6 mice per group.

In order to explore the mechanism by which IL-17 deficient mice transplanted with WT bone marrow had more severe disease in NTN, we sought to identify potential mediators of disease protection. Members of our group have shown that mast cells attenuate glomerulonephritis [24] and mast cells are known to produce IL-17 [25] and to resist radiation [26]. We determined whether mast cells in the kidney could persist and continue to produce IL-17 after irradiation. Bone marrow derived WT C57BL/6 mast cells and splenocytes from Ly5.1 mice were injected beneath the renal capsule of mast cell deficient Kit^W-sh/W-sh^mice. Following irradiation, mast cells unlike splenocytes, survived and were able to produce IL-17. There was no reduction in the number of mast cells in the renal capsule post-irradiation (median (range) IF C-kit non-irradiated mice 116 (80–275); irradiated mice 187 (64–511) p = 0.28) (Fig 6a). In contrast, CD45+ leucocytes were completely removed by irradiation (p = 0.0002) (Fig 6b). Irradiation did not alter the ability of mast cells to produce IL-17 following LPS stimulation (median (range) IF non-irradiated 164 (119–333); irradiated 129 (47–291) p = 0.18) (Fig 6c). These findings are also represented by images from confocal microscopy (Fig 6d).

## Discussion

Through bone marrow transplantation experiments we assessed the relative contributions of IL-17 from haematopoietic and intrinsic cells to NTN, and conclude that (1) transfer of wild-type haematopoietic cells to IL-17 deficient mice restores disease susceptibility and (2) rather than local IL-17 production contributing to pathogenesis as might be expected, it in fact plays a protective role. The WT to IL-17 deficient transplant mice lack intrinsic IL-17, and this resulted in profound kidney injury compared to the WT to WT group. In contrast, WT mice transplanted with IL-17 deficient bone marrow tended to have the mildest disease of the three experimental groups consistent with our observation that local IL-17 production may play a protective role. Renal mast cells may mediate protection in this model since they persist post-irradiation as a source of local IL-17. These data suggest a novel *protective* role for IL-17 produced by intrinsic radio-resistant cells in NTN. To our knowledge, this is the first time that classical bone marrow transplants in IL-17 deficient mice with nephritis have demonstrated restoration of disease susceptibility and the first time that a protective role for IL-17 production by intrinsic cells in glomerulonephritis has been proposed.

The *pathogenic* role for IL-17 and the Th17 axis in autoimmune renal disease has been well established [5–8]. Previous experiments demonstrating restoration of disease susceptibility due to Th17 in NTN have required in vitro polarisation of antigen-specific Th17 cells into strains genetically susceptible to autoimmune disease (Rag^-/-^) with planted antigen bound to the glomerular basement membrane [27, 28]. Our bone marrow transplant experiments provide further convincing evidence for the pathogenicity of haematopoietic IL-17 producing cells in NTN since transplantation of WT bone marrow restores disease susceptibility to protected IL-17 deficient mice.

A *protective* role for IL-17 in renal disease has also previously been proposed. In autologous NTN the role of IL-17 was biphasic in time, pathogenic early on in disease and attenuating disease later, although this did not differentiate between cell compartments [15]. In experimental models of other autoimmune diseases such as graft versus host disease, asthma, Crohn’s disease and uveitis, there is also emerging evidence of a regulatory role for IL-17 [9, 10, 13, 14].

Our results point to a role for intrinsic renal cells in production of protective IL-17. Importantly, levels of renal IL-17 in all transplant groups at the end of the experiment were equal, demonstrating that by day 8 intrinsic and extrinsic sources of IL-17 were comparable and underscoring the importance of local IL-17 production in this model. However, it is also likely that differences in disease outcome between the experimental groups are influenced by differences in levels of IL-17 earlier in the experiment. As described in the previous paragraph, published work has suggested that the role of IL-17 may vary dependent on the time point of disease. In addition, expression of the IL-17 receptor was unchanged between wild-type and IL-17 deficient mice, so more severe disease cannot be explained by upregulation of the IL-17 receptor in IL-17 deficient mice. Apart from T-cells, IL-17 is produced by a number of cell types including mast cells, neutrophils, macrophages, natural killer cells, inducible NKT cells, lymphoid tissue inducer-like cells and γδTcells (reviewed in [29]). The importance of non T-cells such as neutrophils, macrophages and γδTcells in IL-17 production in disease has increasingly been recognised [30–32]. Mast cells in particular are well recognised producers of IL-17 [25, 33]and are known to be resistant to radiation [26], although other immune cells such as macrophages and NK cells have also been shown to persist following radiation [34]. Mast cells can be pathological but also have an important physiological role in homeostasis and tissue repair and a role in immune tolerance [35]. There are conflicting reports of the pathogenicity of mast cells in glomerulonephritis: protective in accelerated NTN [36]and experimental ANCA-associated vasculitis [24] but pathogenic in autologous NTN [37]. We demonstrated that renal mast cells survive irradiation and continue to produce local IL-17 which is protective in accelerated NTN, although other radio-resistant IL-17 producing intrinsic cells such as macrophages might also contribute.

IL-10 is an anti-inflammatory cytokine, and endogenous IL-10 production is known to have a protective effect in NTN [38]. Our experiments demonstrate significantly attenuated levels of IL-10 in the severely affected WT to IL-17 deficient transplant group, and consequently reduced levels of IgG2b. In addition to producing IL-17, mast cells can also produce IL-10 to limit disease [39–41]. It is well recognised that IL-10 can inhibit IL-17 responses [42, 43], so it is conceivable that a local counter-regulatory mechanism could result in mast cells secreting protective IL-17 and stimulating production of IL-10 to limit disease in this model.

Levels of the cytokines IL-1β and IL-23, which are associated with differentiation or maintenance of Th17 cells respectively, were significantly lower in the WT to IL-17 deficient transplant group. Intrinsic cells that produce IL-17 are known to express both the IL-23 and IL-1 receptors, and higher levels of IL-1β and IL-23in the experimental groups with intrinsic IL-17 producing cells may be relevant to lower levels of disease in these groups. Levels of IgG2b were lower, in keeping with the IL-10 results, but otherwise there was comparable glomerular deposition of antibody and humoral anti-sheep IgG systemic immune responses between the experimental groups. This is in keeping with our data from WT and IL-17 deficient mice with NTN and data from other groups in IL-23 deficient mice [6].

In terms of macrophage effector function, lower levels of reparative mannose receptor and arginase associated with alternatively activated macrophages, in the WT to IL-17 deficient transplant group are consistent with more severe disease. iNOS is more usually associated with a pro-inflammatory phenotype so the low level in this experimental group is unexpected, but there is no absolute dichotomy between the multiple macrophage phenotypes now described, which might also vary during the time course of NTN. Numbers of macrophages did not differ between the experimental groups at the end of the experiment and it is likely that earlier variations in macrophage number, or in particular activation state, play a greater role in disease outcome. Our laboratory has previously shown that there may be no difference in macrophage number using this model despite different disease outcomes [44].

Overall, these bone marrow chimeras demonstrate a novel protective role for IL-17 by intrinsic cells in NTN, at least in part likely to be produced by mast cells. A recent observation that radio-resistant follicular dendritic cells in the spleen produce IL-6 and are important in a murine model of chronic viral infection [45]is a precedent for the role of radio-resistant cells in secreting cytokines and modulating disease. This work is timely since blockade of IL-17 or the Th17 axis is being trialled in a number of autoimmune diseases (www.clinicaltrials.gov.uk). Results in two psoriasis trials have been broadly favourable [46, 47]. In contrast, despite evidence from some animal models that IL-17 is implicated in pathogenesis, a trial in Crohn’s disease of secukinumab, a fully human anti-IL-17 monoclonal antibody, was terminated early as it was ineffective with an excess of adverse events [48]. As evidence of a protective role for IL-17 develops, we should be cautious in considering the full implications of clinical IL-17 blockade in autoimmune renal disease.

## Supporting Information

S1 ARRIVE Checklist ARRIVE Guidelines Checklist. (PDF)
